# Artery of Percheron, an Uncommon Variant of Posterior Cerebral Circulation: A Case Report

**DOI:** 10.7759/cureus.57266

**Published:** 2024-03-30

**Authors:** Lalit Ratanpara, Neha Xalxo, Pradip R Chauhan, Simmi Mehra

**Affiliations:** 1 Anatomy, All India Institute of Medical Sciences, Rajkot, Rajkot, IND

**Keywords:** thalamoperforators, posterior cerebral artery, neuroimaging, thalamus, artery of percheron

## Abstract

The posterior communicating artery (PcomA), P1 and P2 segments of the posterior cerebral arteries (PCAs) give rise to numerous small branches that chiefly supply the thalamus and midbrain. Thalamic vascular supply is classically categorized into four regions: anterior, paramedian, infero-lateral and posterior. Despite significant variations and overlap in the blood supply, this traditional classification helps in understanding the vascular anatomy of the thalamus. Gerard Percheron extensively studied thalamic blood supply and described its anatomical variants depending on its origin. The artery of Percheron (AOP) is a rare anatomical variation of paramedian-mesencephalic arterial supply in which a solitary arterial trunk arises from the PCA and distributes bilaterally to both paramedian thalami and often to the rostral part of the midbrain. During routine dissection of the brain of a 46-year-old female in the department of anatomy, it was seen that thalamo-perforating artery (AOP) took origin as a single trunk from the P1 segment of the left PCA. The specimen was dissected and photographed for documentation and to see more details. The exact prevalence of AOP remains unknown, but various studies show that it can be present in 7% to 11.7% of subjects. Detailed knowledge of AOP anatomical variation is crucial for interpreting neuroimaging results or performing different neuro-endovascular techniques at the basilar bifurcation, particularly in patients with bilateral thalamic and midbrain infarctions.

## Introduction

The thalamus serves as a hub for transmitting signals related to both sensory and motor functions and also plays an important role in regulating sleep, alertness, and consciousness. The perforating branches of the P1 and P2 segments of the posterior cerebral artery (PCA) and the posterior communicating artery (PComA) provide a rich blood supply to the thalamus and midbrain [1]. The thalamic vascular supply is traditionally divided into four territories: anterior, paramedian, inferolateral, and posterior, but there may be some variation and overlap. The paramedian area receives its blood supply from the paramedian (or thalamo-perforating) arteries, which arise from the P1 segment of the PCA. The quantity, dimensions, and spatial arrangement of the paramedian arteries supplying the thalamus exhibit notable variations [2].

The artery of Percheron (AOP) originates as a solitary trunk from the thalamic perforating artery, which is the branch of the P1 segment of the posterior cerebral artery and distributes bilaterally to the paramedian thalami and often to the rostral part of the midbrain [3].

Understanding variations in thalamic blood supply is paramount, especially when considering the potential impact on neurological function. Diagnostic challenges associated with AOP infarcts are often overlooked in the initial phases of a CT scan. This oversight can lead to a missed window period ideal for thrombolytic treatment, underlining the critical importance of early recognition. Comprehensive knowledge of perforator anatomy is vital for managing cerebrovascular strokes and surgically treating tumours. Many previous studies highlight that any compromise or injury to these perforators can result in significant neurological deficits [4].

## Case presentation

**Figure 1:**
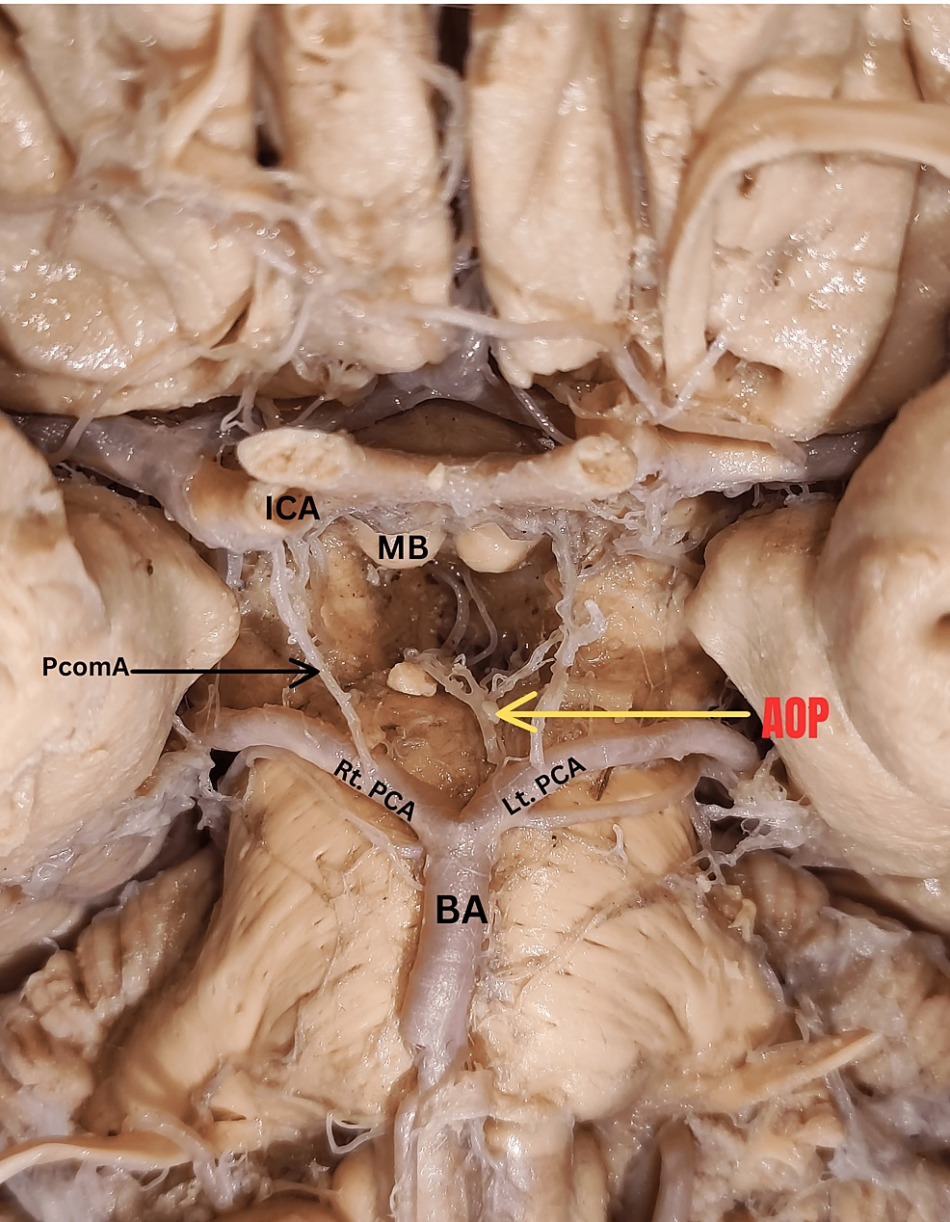
Depicts AOP originating from the P1 segment of left PCA. AOP-Artery of Percheron, BA-Basilar Artery, Lt PCA-Left posterior cerebral artery, Rt PCA- Right posterior cerebral artery, MB- Mamillary body, ICA- internal carotid artery, PcomA- Posterior communicating artery

During routine anatomical dissection of a 46-year-old female brain at the Department of Anatomy, All India Institute of Medical Sciences, Rajkot, Gujarat, it was observed that the basilar artery followed its conventional course, running over the ventral surface of the pons. The basilar artery then bifurcated into the right and left PCAs. Remarkably, a rare but relevant variant was discovered: a solitary trunk originating from the superior aspect of the P1 segment (part of the PCA from its basilar bifurcation to the origin of posterior communicating artery) of the left PCA (Figure 1).

This noteworthy solitary trunk was identified as a variant of thalamo-perforating artery (AOP). This artery coursed upwards for a short distance within the interpeduncular fossa along the ventral surface of the pons, before branching into multiple small arteries. These smaller branches pierced the posterior perforated substance to provide blood supply to both the thalamus and rostral part of the midbrain. Additionally, it was noted that the P1 and P2 segments of the right posterior cerebral artery did not give origin to any thalamo-perforating arteries. The posterior communicating arteries on both right and left sides took origin from the corresponding posterior cerebral arteries and anastomosed with the internal carotid artery of each side respectively to complete the circle of Willis.

## Discussion

Various studies have reported several anatomical variants of the thalamo-perforating branches of the P1 segment of the PCA that supply the paramedian portion of the thalamus. 

In 1966, Westberg initially documented a unique anatomical variation of the thalamo-perforators. This variation involved branches, originating from the P1 segment of the PCA, but was observed only on one side [5]. 

**Figure 2:**
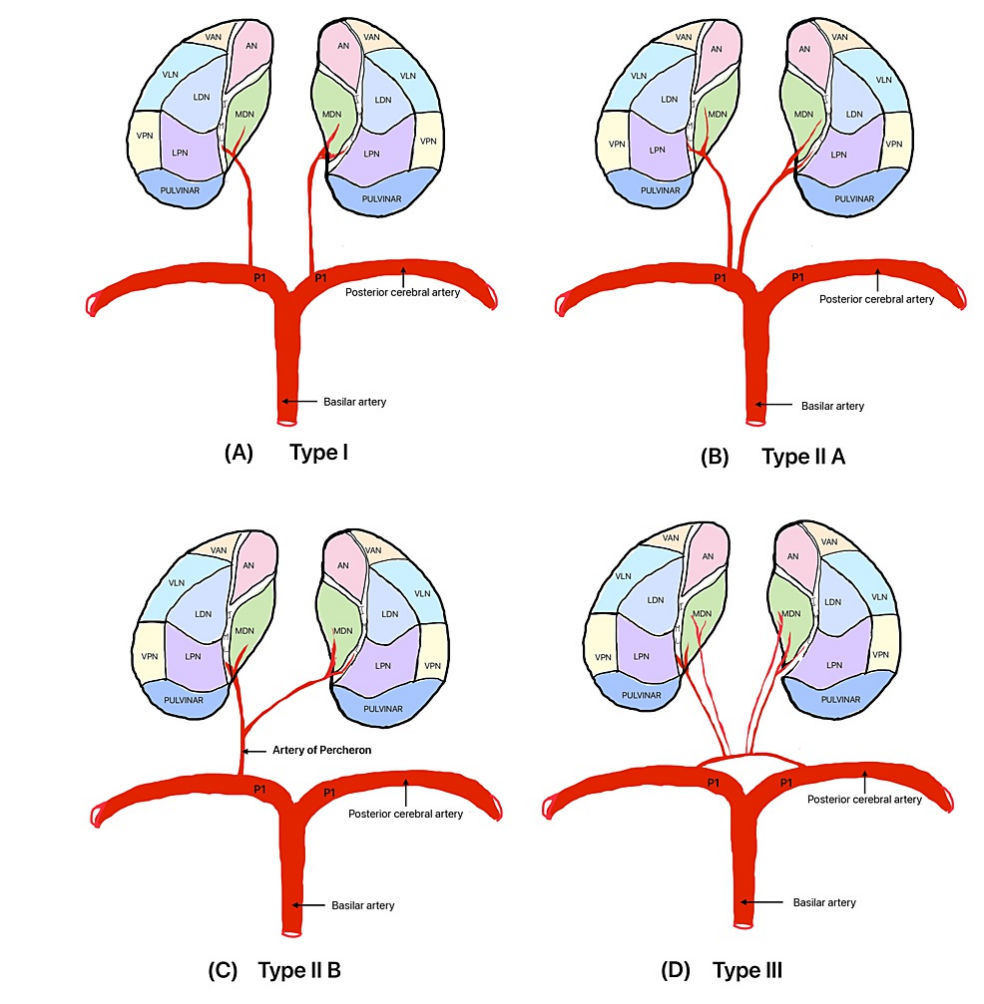
Variants of thalamic blood supply. (A) Type I: Both the left (L) and right (R) paramedian arteries (PMAs) typically originate independently from their respective posterior cerebral arteries (PCAs). This is considered the most common type of thalamic blood supply. (B) Type IIa: Both PMAs emerge from either the left (L) or right (R) PCA, deviating from the usual bilateral origins. (C) Type IIb: The artery of Percheron is a specific type where both thalami are supplied by a single branch that can take origin either from the left (L) or right (R) PCA. (D) Type III: Multiple small perforating branches emanate from a singular arterial arc that spans the P1 segments of both PCAs. AN-anterior nucleus, MDN- mediodorsal nucleus, VAN- ventral anterior nucleus, VLN- ventral lateral nucleus, VPN- ventral posterior nucleus, LPN- lateral posterior nucleus, LDN- Lateral dorsal nucleus (Image credit - Dr. Neha Xalxo)

Gerard Percheron, a French neurologist, conducted thorough research on the blood supply of the thalamus and provided detailed descriptions of its structural variations, which were classified according to their origin [6]. The variations of thalamic blood supply are described in Figure 2.

In one of the anatomical investigations of the posterior circulation, Saeki and Rhoton observed that 8% of cadaveric specimens exhibited a P1 segment without thalamo-perforating branches, but well-developed perforators were noted on the opposite side [7].

Lang and Brunner conducted a study on the perforating branches of the posterior circulation. They discovered that 81% of the inferior posterior diencephalic branches originated from the pars circularis, or P1 segment. In 10% of cases, they identified an anastomosis connecting the right and left sides, which is comparable to Percheron's type III variant, although this study did not explicitly address the frequency of AOPs in their series compared to the previously mentioned research [8].

Pedroza et al. discovered that in 21.5% of the specimens, the superior paramedian mesencephalic and paramedian thalamic arteries took their origin from the solitary P1 segment. 10.7% of cases exhibited the presence of paramedian arteries that originated from a single trunk, similar to the AOP configuration [9].

In another study by Uz, 15 cadaveric brains were examined, and they discovered that in 7% of the specimens, either a single large or several small arteries originated from the P1 segment on one side and supplied the thalamus bilaterally [10]. In the study by Park et al., a unilateral, solitary P1 perforator was discovered in 11.5% of the cases [11].

Kocaeli et al. examined 68 posterior cerebral arteries in 34 preserved cadaveric brains. They noted that the thalamo-perforating arteries originated from the upper or back surface of the P1 segment of PCA and entered the brain through the posterior perforated substance. In addition, they documented an incidence rate of 11.7% of AOP where no branches were seen on the opposite side [12].

Griessenauer et al. identified AOP in 12% of specimens out of 25 adult latex-injected cadaveric brains. In two cases, the artery was observed on the right side whereas it was on the left side in one specimen. It arose lateral to the basilar artery bifurcation, which is in line with the current case report [13].

The largest case series to date was published by Lazzaro et al. and consisted of 37 cases that were used to characterize the entire imaging spectrum of AOP infarctions. Clinically, altered state of consciousness, vertical gaze palsy, and impaired memory are the traditional signs of a thalamic paramedian infarction [14].

The incidence rate of artery of Percheron infarction ranges from 0.1% to 0.3%. The various and complex anatomical features and varying vascularity are the primary factors contributing to the different clinical manifestations of thalamic infarcts. Diagnosing the artery of Percheron infarction can be challenging due to its diverse morphological manifestations and the variability in size of the P1 segment. When necessary, it is important to rule out potential differential diagnoses such as Japanese encephalitis, Wernicke's encephalopathy, and cerebral venous sinus thrombosis, which may present with bilateral thalamic lesions. MRI diffusion-weighted imaging (DWI) and fluid attenuated inversion recovery (FLAIR) MRI sequences are the preferred methods for promptly diagnosing AOP infarction [15].

## Conclusions

The consideration of AOP variation is imperative when encountering paramedian thalamic infarction in neuroimaging, despite its rarity. As conventional angiography typically does not visualize AOP, comprehension of its variations is pivotal for accurate imaging interpretation, particularly in cases of bilateral paramedian thalamic and midbrain infarction. Furthermore, understanding AOP anatomy is essential in surgical or neuro-endovascular interventions involving the basilar bifurcation. In cases of aneurysm treatment where AOP is present, an ipsilateral approach is recommended to ensure optimal visualization, preservation, and mitigate thrombo-embolic risks during endovascular procedures targeting basilar apex aneurysms. Although this anatomical variation may not inherently signify pathology, surgeons must acknowledge this anomaly to mitigate potential complications and iatrogenic injuries during surgical interventions.
